# Advances in nutritional metabolic therapy to impede the progression of critical illness

**DOI:** 10.3389/fnut.2024.1416910

**Published:** 2024-07-05

**Authors:** Wenwei Chen, Jia Song, Shijin Gong

**Affiliations:** ^1^The Second School of Clinical Medicine, Zhejiang Chinese Medical University, Hangzhou, China; ^2^Zhejiang Hospital, Hangzhou, China

**Keywords:** chronic critical illness (CCI), critically ill, review, nutrition support, metabolic

## Abstract

With the advancement of medical care and the continuous improvement of organ support technologies, some critically ill patients survive the acute phase of their illness but still experience persistent organ dysfunction, necessitating long-term reliance on intensive care and organ support, known as chronic critical illness. Chronic critical illness is characterized by prolonged hospital stays, high mortality rates, and significant resource consumption. Patients with chronic critical illness often suffer from malnutrition, compromised immune function, and poor baseline health, which, combined with factors like shock or trauma, can lead to intestinal mucosal damage. Therefore, effective nutritional intervention for patients with chronic critical illness remains a key research focus. Nutritional therapy has emerged as one of the essential components of the overall treatment strategy for chronic critical illness. This paper aims to provide a comprehensive review of the latest research progress in nutritional support therapy for patients with chronic critical illness.

## Definition of chronic critical illness

The term Chronic Critical Illness (CCI) was first introduced by Girard (1) in 1985 to describe a group of patients who have survived the acute phase of their illness but continue to experience persistent organ dysfunction, necessitating prolonged reliance on intensive care and organ support (2). This condition is an inevitable outcome of modern medical advancement. With the progress in healthcare standards and the exacerbation of population aging, the prevalence of CCI is on the rise globally, particularly in high-income countries (3), garnering significant attention in the academic community.

Previously, CCI has been described using various terms such as critical illness polyneuropathy, critical illness myopathy, ICU-acquired weakness, and post-intensive care syndrome (4–7). Gardner et al. (8). proposed the latest diagnostic criteria for CCI, defined as an ICU stay exceeding 14 days with ongoing organ dysfunction on the 14th day (SOFA score ≥ 1 or any other organ system score ≥ 2).

### Epidemiology of chronic critical illness

Although the incidence of CCI is increasing year by year, its epidemiological characteristics are still unclear, and there is limited reliable information about the incidence and outcomes of this syndrome. Previous epidemiological assessment data only come from a few hospitals and limited geographic regions, lacking strong representativeness. Previous studies have also used different definitions of CCI, reflecting a lack of consensus on this complex syndrome.

Kahn et al. (9). conducted a retrospective cohort study using discharge data from five states in the United States from 2004 to 2009. The inclusion criteria for CCI were ICU stay ≥8 days and meeting at least one of the following conditions: (1) mechanical ventilation; (2) tracheostomy; (3) sepsis and other severe infections; (4) severe trauma; (5) stroke (including ischemic stroke and intracerebral hemorrhage). In the study sample, CCI accounted for 7.6% of all ICU admissions, with an overall population incidence rate of 34.4 per 100,000. The incidence rate of elderly chronic critical illness patients aged 65 and above gradually increased with age, peaking at 75–79 years, reaching 82.1 per 100,000. This indicates that chronic critical illness is particularly common in the elderly, although the incidence decreases in the population aged 80 and above, partly due to early death (within 8 days).

Ohbe et al. (10). used hospitalization data from 679 hospitals in Japan from 2011 to 2018, using the same CCI diagnostic criteria. Similar to the results of Kahn’s study, the overall population incidence rate of CCI was 42.0 per 100,000, and the prevalence of elderly chronic critical illness increased steadily with age, reaching 109.6 per 100,000 in the population aged 85 and above.

The above results indicate that the incidence of CCI sharply increases with age, posing a threat not only to patients’ health but also imposing a heavy burden on their families, healthcare systems, and society due to the significant utilization of ICU resources (8). However, despite such huge expenses, the prognosis of CCI patients remains poor. The in-hospital mortality rate is 30% (9), and the 5-year mortality rate is approximately 81%, much higher than most malignancies (20–49%) (11). Therefore, research also emphasizes the necessity of preventing CCI and reducing related care costs. In recent years, with the development of nutritional support technology, nutritional therapy has become one of the strategies used in the overall treatment of elderly chronic critical illness.

### Characteristics of nutritional metabolism in chronic critical illness

In 2019, the Sepsis and Critical Illness Research Center in the United States proposed a theoretical framework for CCI centered around damage-associated molecular patterns (DAMPs). This theory elucidates the molecular mechanisms underlying the development of CCI. DAMPs are non-microbial molecules released from cells upon tissue damage, effectively activating the immune system and initiating sustained non-infectious inflammatory responses. This process results in systemic inflammation, organ damage, and potentially mortality. Analogous to other endogenous alarm signals, an increase in local tissue damage and systemic inflammation leads to sustained inflammatory responses (12). Some studies indicate that muscle breakdown metabolism can result in the release of DAMPs (including mtDNA, HMGB1, and TFAM) into the systemic circulation, thus perpetuating inflammation and impacting patient prognosis (13).

Currently, the academic community considers Persistent Inflammation Immunosuppression Catabolism Syndrome (PICS) as the primary pathophysiological mechanism underlying CCI (14, 15). Prolonged inflammation leads to abnormal myelopoiesis (myeloid-derived suppressor cells, MDSCs), T cell atrophy, T cell exhaustion, and expansion of inhibitory cell function.

Furthermore, sustained inflammation and oxidative stress impact nutritional status, resulting in anorexia, reduced food intake, and muscle catabolism (16, 17). The interplay between malnutrition, oxidative stress, and inflammation exacerbates muscle loss, affecting muscle structure, and increasing the risk of sarcopenia and weakness (18, 19). Moreover, autophagy deficiency is recognized as another mechanism underlying critical illness. Autophagy, a selective cellular process for organelle quality control, facilitates the clearance of damaged organelles and molecules. Although autophagy typically occurs post-muscle injury and is pivotal for muscle function recovery, its activation appears inadequate in critical illness, despite various triggers such as hypoxia, oxidative stress, and endoplasmic reticulum stress, thereby exacerbating muscle atrophy (20). Consequently, elderly individuals with chronic critical illness are particularly susceptible to malnutrition, sarcopenia, weakness, and autophagy deficiency, precipitating disease progression.

The metabolic characteristic of CCI is marked protein breakdown, which is due to decreased protein synthesis, uncontrolled muscle breakdown, and the release of potential pro-inflammatory degradation products (21, 22). Insulin resistance and breakdown reactions lead to the consumption of energy stores such as liver glycogen (glucose), fat (free fatty acids), and muscle (amino acids) (23). Within a few days of admission, protein breakdown in critically ill patients can reach nitrogen levels of 12–16 g/day, and in some cases, it can increase to nitrogen levels of 30 g/day (24). Previous studies have shown that muscle protein synthesis capacity in the elderly decreases by approximately 10–20% (25), it has been proposed that the diminished response to protein intake on muscle protein synthesis (MPS) in the elderly may be a contributing factor (26). Which has been confirmed in frail populations (27, 28). This is undoubtedly a vicious cycle for elderly chronic critical illness patients, leading to severe cachexia.

Based on the pathophysiology of CCI, adequate nutrition can potentially prevent it by modulating immunity, supporting autophagy, and preventing muscle catabolism.

### Assessment indicators of nutritional metabolism

Sarcopenia, weakness, and malnutrition often reciprocally influence each other. Sarcopenia manifests as progressive and widespread loss and alterations in skeletal muscle mass and strength. As a component of catabolic syndrome, sarcopenia is prevalent in the ICU, particularly among elderly critically ill patients.

Various imaging assessment methods exist for sarcopenia, which is regarded as one of the nutritional metabolic indicators. Ultrasonography (US) is a portable, non-invasive, and ionizing radiation-free technique extensively employed in musculoskeletal studies. It exhibits a robust positive correlation with CT and MRI; however, most investigations concentrate on the quadriceps femoris muscle, with limited exploration of other muscles like the psoas muscle, and ultrasound outcomes are subject to operator proficiency. CT, serving as a standard diagnostic tool for assessing muscle quantity and quality, boasts advantages in disease staging and longitudinal monitoring, characterized by reduced variability and standardized measurements. MRI offers insights into muscle composition via diverse sequences, delivering superior soft tissue imaging, particularly for muscles, compared to CT, albeit at a higher cost (29).

Furthermore, bioelectrical impedance analysis (BIA), a practical and portable technique for estimating body composition through predictive equations, involves applying low-intensity currents to the body to measure the impedance of fluids within and outside cells, as well as the capacitive component reflecting the cell membrane’s impedance. However, BIA’s reliability is significantly affected by factors such as fever and fluid shifts, particularly edema, which are prevalent in critically ill patients (30, 31). Currently, the devices employed for BIA are not widely available for clinical use (29).

In addition to imaging indicators, clinical assessment commonly incorporates physiological indicators to comprehensively evaluate the nutritional metabolism of chronic critically ill patients. With increased breakdown metabolism in elderly chronic critically ill patients resulting in elevated muscle breakdown, urea, a metabolic byproduct, also rises. Conversely, creatinine production diminishes with decreasing muscle mass. In a heightened breakdown state, the urea to creatinine ratio (UCR) increasingly reflects the characteristics of breakdown metabolism in critical illness.

Volbeda et al. (32). discovered that patients hospitalized in the ICU for 30 days or more exhibited a daily decrease in urinary creatinine excretion of 1% or more, with the urea to creatinine ratio (UCR) as an indicator of breakdown metabolism showing a continuous increase during the first 30 days of ICU admission. Zhang et al. (33). conducted a retrospective cohort study indicating that among septic patients in the ICU, those with Persistent Critical Illness (PCI) experienced more significant changes in UCR compared to non-PCI patients, notably increasing from day 4 to day 10. Despite limitations in the database, such as the absence of reasons for prolonged ICU stays, this data provides compelling evidence of biochemical markers of chronic critical illness. In 2021, Flower et al. (34). examined the impacts of intermittent versus continuous feeding on UCR, revealing that intermittent enteral nutrition may mitigate the rise in UCR among critically ill patients. Although urea and creatinine levels are subject to influences from heart failure, dehydration, upper gastrointestinal bleeding, and acute kidney injury, their ratio remains less impacted. Additionally, UCR serves as a discriminant for patients with Persistent Critical Illness, PICS, and postoperative muscle atrophy (35, 36).

In addition to UCR, clinical indicators utilized to ascertain heightened breakdown metabolism encompass serum albumin levels below 30 g/L, prealbumin levels under 100 mg/L, creatinine height index below 80%, weight loss exceeding 10% during hospitalization, or a BMI below 18 kg/m^2^ (37). Nevertheless, these indicators, such as albumin and prealbumin levels, prove unreliable owing to the influence of inflammation, heightened vascular permeability, and plasma protein leakage, which fail to accurately portray the patient’s metabolic state (38). Consequently, there exists an imperative to devise novel biomarkers for early monitoring of breakdown metabolism in critically ill patients.

### Nutritional support

#### Enteral nutrition (EN)

Chronic critically ill patients often encounter difficulties with oral intake, wherein nutritional support assumes a pivotal role, encompassing both enteral and parenteral nutrition. Typically, CCI patients opt for enteral nutrition (EN), which can ameliorate nutrition and overall condition, safeguard the integrity of the intestinal mucosa, uphold intestinal barrier function, mitigate inflammatory responses, and bolster immune function. It proves advantageous in diminishing the risk of complications such as gastrointestinal infections and ventilator-associated pneumonia, thereby positively influencing disease outcomes (39–41). Relative to parenteral nutrition support, enteral nutrition manifests as more physiologically compatible.

European Society for Clinical Nutrition and Metabolism (ESPEN) guidelines underscore the importance of initiating early enteral nutrition (EEN) within 48 h if patients are unable to orally intake food, barring instances of uncontrolled shock, hypoxemia, or acidosis. The fundamental principle is rooted in the observation that alterations in the intestinal barrier can manifest within 24 h, evidenced by signs of intestinal ischemia, heightened permeability, bacterial translocation, and dysbiosis (42). Concerning the total energy provision via enteral feeding, the American Society for Parenteral and Enteral Nutrition (ASPEN) contends that in adult critically ill patients, there exists no significant disparity in patient outcomes between high-energy and low-energy intake. It is recommended to administer 12–25 kcal/(kg·d) of energy during the initial 7–10 days in the ICU (40). Nonetheless, the European Society for Clinical Nutrition and Metabolism (ESPEN) advocates employing methods such as indirect calorimetry (IC), pulmonary artery catheter VO2 assessment, predictive equations, etc., to tailor nutrient intake recommendations to each patient’s specific needs.

However, it should be recognized that both overfeeding and underfeeding are associated with poor outcomes, and the study by Zusman et al. (43). reached a similar conclusion that both overfeeding and underfeeding are harmful. Critical patients have significant changes in daily energy expenditure (44–46), and the optimization of nutritional support is hampered at both the group and individual levels due to a lack of observations of changes in nutrient requirements during ICU stay in critically ill patients. Among them, IC is a clinical measurement method used to measure energy expenditure (EE) and monitor patients’ energy fluctuations, and optimize energy prescription with a personalized medicine orientation (47).

Both ESPEN and ASPEN tend to use IC to assess a patient’s actual energy expenditure. The prediction equation is associated with significant inaccuracies, leading to over- or underestimation of demand and inducing over- or under-feeding (48). In the early stages of the disease, low-calorie nutrition (no more than 70 percent of the EE) should be given, but after the third day, energy delivery can increase to 80 to 100 percent of the EE, reflecting a gradual increase in energy supply (42).

Intolerance to enteral nutrition poses a significant challenge to its smooth progression, with an average incidence of 33%. This issue is especially prevalent in mechanically ventilated patients in the ICU, with an incidence as high as 80.2–85.0% (49). Gastrointestinal intolerance not only causes discomfort but also frequently interrupts enteral nutrition, hindering the achievement of target supply volumes. Only about 40–60% of patients reach recommended nutritional targets (50). The delivery mode of enteral nutrition, whether continuous or intermittent, is a subject of debate. Some studies suggest that continuous feeding may reduce diarrhea and aspiration (51). Hong-Yeul et al. (52). conducted an RCT comparing intermittent and continuous enteral feeding, with the continuous group achieving significantly higher nutritional targets (≥80%) than the intermittent group. However, intermittent EN may facilitate intermittent secretion of gastrointestinal hormones, which supports the physiological environment of gastrointestinal digestion and absorption. As mentioned earlier, autophagy deficiency, which eating can activate and nutritional supply can reverse, may be exacerbated by continuous feeding in critically ill patients, although the specific mechanism remains unclear (20). Therefore, more high-quality RCTs and basic research are necessary to validate the efficacy and safety of continuous versus intermittent feeding.

#### Parenteral nutrition(PN)

Parenteral nutrition (PN) is a form of intravenous nutritional support that circumvents the gastrointestinal system. When enteral nutrition proves insufficient or impractical, PN serves as a commonly employed alternative to ensure adequate nutrition. PN comprises amino acids, glucose, and lipids to fulfill energy and protein requirements. Its primary drawback lies in infectious complications, as traditionally believed, with PN linked to an elevated overall infection risk, including pneumonia and intra-abdominal abscesses (53). Elke et al.’s (54) meta-analysis of 18 RCTs compared clinical outcomes between enteral and parenteral nutrition in ICU patients. The infection rate significantly differed between the EN and PN groups, particularly notable in patients with notably increased calorie intake in the PN group (54). Thus, it can be inferred that calorie overload (and overfeeding) heightens infection risk, rather than PN *per se*. Jean et al.’s RCT also supports this notion: in ICU settings, when PN and EN provide equal energy, clinical outcomes, including mortality and complications, exhibit no significant variance between the groups (55). The latest ASPEN guidelines advocate initiating PN when gastrointestinal tract contraindications or inadequacies hinder nutrient delivery, rather than postponing EN.

PN can function as either a sole source of nutrition or an additional source (supplemental PN, SPN) when oral intake or EN proves insufficient to meet complete nutritional needs. The latest ASPEN guidelines (40) underscore that for critically ill patients, comparable energy intake can be achieved through PN or EN during the initial week of illness, with no substantial disparities in clinical outcomes. Consequently, short-term PN supplementation is deemed safe, efficacious, and yields outcomes akin to EN. Nonetheless, ASPEN contends that early supplemental parenteral nutrition does not confer significant benefits and advocates commencing SPN after 7 days in the ICU.

The composition of parenteral nutrition formulations significantly impacts patient prognosis. Andrew et al.’s study revealed that compared to adequate fat-high glucose (ALHD) nutrition, patients receiving high-fat-low-glucose (HLLD) parenteral nutrition experienced a 42.6% decrease in CRP levels ([11.5 ± 6.8] vs. [6.6 ± 6.0]; *p* = 0.028), a 40.6% increase in prealbumin levels ([13.0 ± 5.8] vs. [21.9 ± 8.6]; *p* < 0.001), and a 64.1% decrease in the incidence of blood glucose levels >180 mg/dL ([35.1 ± 31.2] vs. [12.6 ± 21.8]; *p* = 0.003) while maintaining similar calorie targets (56). However, discussions on the types of nutritional formulations are less prevalent in chronic critically ill patients, and further randomized controlled trials (RCTs) are necessary in the future to inform the use of parenteral nutrition in this population.

### Protein supplementation and immunonutrients

Proteins play a pivotal role during critical illness. In chronic critically ill patients, diminished synthetic metabolism, insulin resistance, inflammation, and reduced satellite cell count contribute to weakened synthetic metabolic responses (38). Simultaneously, the body shifts towards catabolic metabolism, with proteins rapidly breaking down as the primary energy substrate, leading to heightened infectious complications and prolonged recovery (57). However, it is important to note that protein intake in the first week of the ICU is not associated with loss of muscle mass (58).

Furthermore, sarcopenia is prevalent among critically ill elderly populations and may exacerbate during hospitalization (59), resulting in significant immobilization in ICU settings. Muscle wasting can be classified into qualitative or quantitative loss, with quantitative depletion occurring due to alterations in protein metabolism, increased amino acid oxidation, reduced caloric and protein intake, inflammatory status, diminished peripheral perfusion, and prolonged immobility, leading to both a quantitative decline and compromised muscle quality (60). Zudin et al. (61) reported a significant decrease in the cross-sectional area of the quadriceps femoris in critically ill patients, with a 12% drop in the first week and a more substantial decline of up to 17.7% by day 10.

Therefore, administering exogenous protein stands as the most direct approach to offsetting protein loss in the body. Nevertheless, controversy persists regarding the optimal dosage of protein supplementation. Several large observational studies have indicated that higher protein intake correlates with improved survival rates (62).

Recent studies, such as PROTINVENT (63), have revealed a higher mortality rate among patients treated with high protein doses during the first 3 days. The EPaNIC post-hoc analysis underscores the potential negative impact of excessive nutrition, as it can disrupt autophagy, thereby hindering the degradation of damaged cellular proteins and organelles, which in turn prolongs organ dysfunction (64). Recent findings indicate that the conversion rate of amino acids improves over time, with a significant increase in overall protein synthesis occurring during the acute phase’s early stages and persisting even after (65, 66). This body of evidence supports the notion of a gradual increase in protein intake, as low and gradual dosages of protein can prevent excessive consumption during periods of heightened endogenous energy production, while also mitigating the risk of refeeding syndrome by suppressing autophagy (67, 68).

Numerous studies tend to link higher protein provision with lower incidence and mortality rates compared to lower protein intake levels (69, 70). However, current evidence does not unequivocally support the notion that elevated average protein intake [1.31 ± 0.48 vs. 0.90 ± 0.30 g/(kg·d)] confers benefits for critically ill patients (71). The latest ASPEN guidelines (40) suggest that, due to a paucity of high-quality evidence trials, adherence to the 2016 guidelines recommending an intake of 1.2–2.0 g/(kg·d) of protein is prudent. Similarly, ESPEN (42) has issued analogous recommendations, indicating no significant disparities in outcomes between protein intakes of 1.6 ± 0.5 g/(kg·d) and 0.9 ± 0.3 g/(kg·d), albeit higher protein intake has been associated with increased mortality in acute kidney injury patients. Some studies propose a protein intake of 2.5 g/(kg·d) for critically ill elderly patients in a hypercatabolic state (72), thus, a unified standard for protein intake in elderly chronic critically ill patients remains elusive.

In addition to proteins, various immunonutrients such as arginine and leucine exert a beneficial impact on the prognosis of critically ill patients. Arginine, beyond its typical amino acid functions, also acts as a substrate for intracellular nitric oxide (NO) and is intricately linked to patients’ immune function. A study suggests that supplementing arginine may counteract the continuous arginine deficiency caused by the persistent expansion of MDSCs during PICS, thereby promoting lymphocyte proliferation and enhancing tissue repair (73). It can also improve muscle strength and physical function in older adults with low protein consumption, frailty, and sarcopenia, and increase postprandial MPS (74). Furthermore, supplementation of leucine and other branched-chain amino acids (BCAAs) can stimulate protein synthesis and inhibit protein breakdown via the mTOR pathway, thereby enhancing nutritional and immune parameters such as nitrogen balance, prealbumin levels, and lymphocyte counts (3, 24). For critically ill elderly patients, supplementation with immunomodulators like ω-3 fatty acids and γ-linolenic acid can significantly reduce mechanical ventilation duration and mortality risk (75).

### Application of probiotics

Throughout critical illness, epithelial cells and the intestinal mucosal barrier undergo changes, resulting in alterations in the abundance and diversity of gut microbiota, a phenomenon termed dysbiosis (76). ICU patients demonstrate notable shifts in gut microbiota diversity compared to healthy individuals, marked by a reduction in obligate anaerobes and an elevation in pathogenic bacteria, predisposing them to complications such as sepsis and systemic inflammatory response syndrome (SIRS) (77).

As previously mentioned, CCI patients often experience prolonged inflammatory responses, necessitating high doses of steroids or antibiotics to mitigate inflammation, which further impairs the body’s bactericidal and bacteriostatic capacities. This phenomenon results in intestinal and mesenteric lymph node enlargement, disrupting the intestinal microecological environment and leading to disorders in intestinal absorption, infections (78, 79), and increased susceptibility to hospital-acquired infections, sepsis, multiple organ dysfunction syndrome (MODS), energy imbalance, muscle wasting, and cachexia (80). This situation is exacerbated in elderly chronic critically ill populations. Victoria et al. (81). investigated the fecal microbiota profiles of elderly ICU patients and discovered that compared to younger individuals, patients aged over 60 exhibited lower bacterial diversity and higher pathogen abundance in the ICU, including genera such as Escherichia-Shigella and Hungatella. Hence, elderly individuals are more susceptible to dysbiosis, underscoring the growing significance of microbial modulators.

Microbial modulators play a crucial role in regulating immune dysfunction, enhancing local immune function, preventing damage to the intestinal mucosal barrier, maintaining gut microbiota balance, and promoting gastrointestinal motility and absorption. Studies have shown (82, 83) that probiotics, such as lactobacilli, bifidobacteria, and *Lactobacillus acidophilus*, can colonize various parts of the intestine, thereby preventing invasion by pathogenic microorganisms, supplementing dominant bacteria, assisting in vitamin synthesis, scavenging free radicals, regulating the intestinal microenvironment and circulation, and promoting nutrient absorption.

Zhang et al. (84). randomly divided in-hospital chronic critically ill (CCI) patients into two groups. The intervention group received microbial modulators in addition to enteral nutrition, while the control group received only enteral nutrition. The results indicated that serum total protein ([69.75 ± 7.48] vs. [62.70 ± 6.33]) g/L, albumin ([38.91 ± 3.54] vs. [34.83 ± 3.82]) g/L, and prealbumin ([204.24 ± 28.80] vs. [187.64 ± 23.73]) mg/L were higher in the intervention group than in the control group (*p* < 0.05). Therefore, the inclusion of microbial modulators has a beneficial effect on nutritional improvement in elderly chronic critically ill patients.

### Exercise and utilization of anabolic-androgenic steroids (AAS)

It is well known that exercise improves muscle strength and function and reduces inflammation and oxidative stress, and that certain physical activity can stimulate MPS, with significant increases in aerobic training (AT) and resistance training (RT) regimens (85, 86), de Azevedo et al. (87). conducted a randomized controlled trial, where critically ill patients were allocated to either a high-protein supplementation with early exercise intervention group or a control group. Both groups employed the IC to monitor EE, with a gradual escalation to 80% of their total energy output. The study’s findings revealed that after 3 and 6 months, the high-protein group, which engaged in resistance training, exhibited significantly higher Physical Component Summary (PCS) scores compared to the control group. Despite the presence of synthetic metabolic resistance that impedes the capacity of RT to achieve a constant positive protein balance throughout the day (88), the combination of high protein intake and early exercise has been proven to significantly enhance the prognosis of critically ill patients. Furthermore, the ongoing Nexis trial by Heyland et al. (89). is examining the combined impact of early intravenous amino acid supplementation and bed-based cycle power testing exercise on patient outcomes, marking it as the first randomized controlled trial to assess the synergistic effects of exercise and protein supplementation in the early phase of critical illness.

Therefore, early physiotherapy in the ICU may prevent or reverse physical damage. Kayambu et al. (90). investigated the effect of exercise on clinical outcomes in critically ill patients using randomized controlled trials, meta-analysis, and systematic reviews, and showed that supporting physical therapy in the ICU had a significant positive effect on improving peripheral and respiratory muscle strength, quality of life, physical function, increasing ventilator-free days, and reducing hospital and ICU hospital stays. However, there was no significant positive effect on mortality. Cheryl et al. (91). showed that critically ill patients who underwent early rehabilitation physiotherapy had a significantly higher number of muscle fibers and an increase in muscle fiber thickness, which was positively correlated with daily activity compared with control groups. Therefore, for CCI patients, early exercise physical rehabilitation is very important.

One approach to enhance muscle mass in critically ill patients is through creatine supplementation, which increases the availability of creatine and phosphocreatine in muscles, thereby promoting insulin-like growth factor 1 (IGF-1) expression and protein phosphorylation, supporting anabolic metabolism (92). It has been shown to improve muscle quality and enhance exercise capacity in older adults, and when combined with resistance training, it leads to greater adaptive responses in skeletal muscle compared to standalone training. For athletes, long-term supplementation with a combination of creatine 3–10 g/day and calcium β-hydroxy-β-methylbutyrate (HMB) 3 g/day has been shown to have a positive effect on athletic performance and body composition in athletes (93), However, there is currently no specific research focusing on creatine supplementation for critically ill patients.

Furthermore, supra-physiological doses of anabolic steroids have been shown to enhance muscle strength in otherwise healthy individuals. A catabolic response in the ICU can be counteracted with anabolic therapies (93). Testosterone, through its androgenic pathway, can minimize muscle wasting and autophagy, as evidenced in severe burn patients. Anabolic agents like oxandrolone and IGF-1 have also been found to reduce muscle catabolism in burn victims, thereby mitigating the progression of PICS. In addition, medications like IL-1 and IL-6 receptor antagonists can alleviate chronic inflammation, but the timing of administration is highly contentious due to the risk of stimulating or inhibiting other pertinent signaling pathways if not appropriately employed (94).

### Summary and outlook

**Figure 1:**
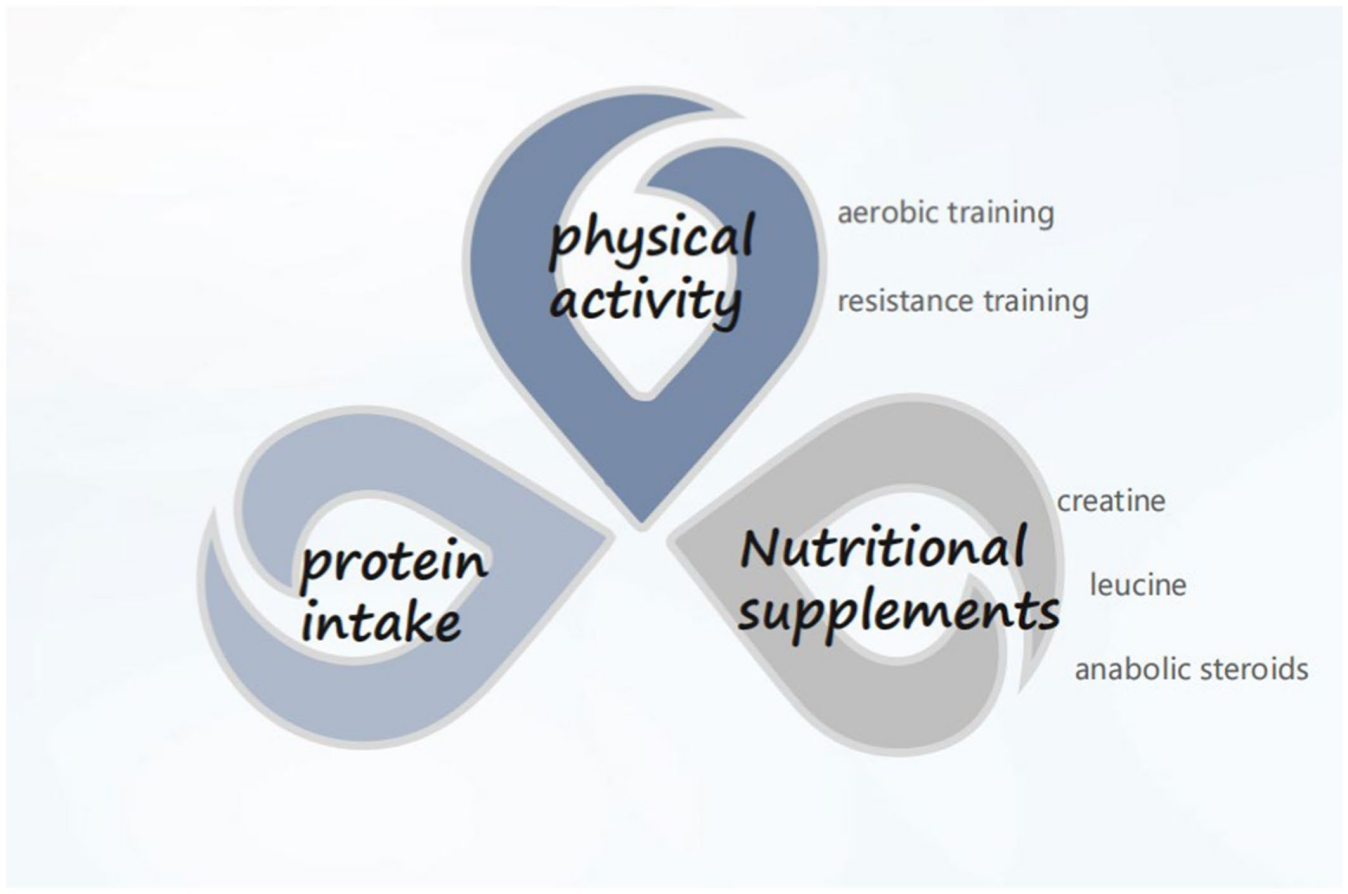
Available methods for overcoming anabolic resistance (adapted with permission from Panda).

CCI is garnering increasing attention, with clinical nutrition emerging as an integral component of treating critically ill patients. Presently, priority is accorded to oral intake for critically ill patients. In cases where oral intake is not feasible, early initiation of enteral nutrition support is recommended. In the presence of contraindications to enteral nutrition, timely provision of parenteral nutrition should be considered rather than discontinuing nutrient intake. Additionally, it is advisable for ICU patients to incorporate an appropriate amount of probiotics into their regimen to ameliorate gut microbiota dysbiosis. As far as the pathogenesis of CCI is concerned, improving the process of PICS and alleviating the anabolic resistance of patients can be started from protein intake, nutritional supplementation (essential amino acids, creatine, etc.), and exercise (aerobic exercise, resistance training) (Figure 1). Artificial feeding has transitioned from being an alternative treatment modality to a therapy requiring diligent oversight and monitoring. Similar to other therapeutic approaches, effective monitoring is essential to ensure safety and achieve desired outcomes. Particularly in critically ill patients, including the elderly, frail, and malnourished, real-time feedback on nutrition delivery proves advantageous for physicians in determining the most suitable feeding goals and methods for patients. However, research on nutrition in elderly chronic critically ill populations remains limited. It is hoped that future randomized controlled trials will yield further insights to inform nutritional support strategies for these patients.

## Author contributions

WC: Visualization, Writing – original draft, Writing – review & editing. JS: Writing – review & editing. SG: Project administration, Supervision, Writing – review & editing.
